# Impaired arterial vitamin D signaling occurs in the development of vascular calcification

**DOI:** 10.1371/journal.pone.0241976

**Published:** 2020-11-19

**Authors:** Kenneth Lim, Guerman Molostvov, Maria Lubczanska, Simon Fletcher, Rosemary Bland, Thomas F. Hiemstra, Daniel Zehnder

**Affiliations:** 1 Division of Nephrology, Department of Medicine, Indiana University School of Medicine, Indianapolis, IN, United States of America; 2 Institute of Cancer and Genomic Sciences, College of Medical and Dental Sciences, University of Birmingham, Birmingham, United Kingdom; 3 Divisions of Biomedical Sciences, University of Warwick, Coventry, United Kingdom; 4 Department of Nephrology, University Hospital Coventry and Warwickshire NHS Trust, Coventry, United Kingdom; 5 Cambridge Clinical Trials Unit and School of Clinical Medicine, University of Cambridge, Cambridge, United Kingdom; 6 Department of Nephrology and Department of Acute Medicine, North Cumbria Integrated Care University Hospital NHS Trust, Carlisle, United Kingdom; University of Texas Southwestern Medical Center, UNITED STATES

## Abstract

Conflicting data exists as to whether vitamin D receptor agonists (VDRa) are protective of arterial calcification. Confounding this, is the inherent physiological differences between human and animal experimental models and our current fragmented understanding of arterial vitamin D metabolism, their alterations in disease states and responses to VDRa’s. Herein, the study aims to address these problems by leveraging frontiers in human arterial organ culture models. Human arteries were collected from a total of 24 patients (healthy controls, n = 12; end-stage CKD, n = 12). Cross-sectional and interventional studies were performed using arterial organ cultures treated with normal and calcifying (containing 5mmol/L CaCl_2_ and 5mmol/L β-glycerophosphate) medium, *ex vivo*. To assess the role of VDRa therapy, arteries were treated with either calcitriol or paricalcitol. We found that human arteries express a functionally active vitamin D system, including the VDR, 1α-hydroxylase and 24-hydroxylase (24-OHase) components and these were dysregulated in CKD arteries. VDRa therapy increased VDR expression in healthy arteries (p<0.01) but not in CKD arteries. Arterial 1α-OHase (p<0.05) and 24-OHase mRNA and protein expression were modulated differentially in healthy and CKD arteries by VDRa therapy. VDRa exposure suppressed Runx2 and MMP-9 expression in CKD arteries, however only paricalcitol suppressed MMP-2. VDRa exposure did not modulate arterial calcification in all organ culture models. However, VDRa reduced expression of senescence associated β-galactosidase (SAβG) staining in human aortic-smooth muscle cells under calcifying conditions, *in vitro*. In conclusion, maladaptation of arterial vitamin D signaling components occurs in CKD. VDRa exposure can exert vasculo-protective effects and seems critical for the regulation of arterial health in CKD.

## Introduction

Arterial calcification is a major contributor to cardiovascular disease in patients with Chronic Kidney Disease (CKD) [1]. Arterial calcification occurs at an accelerated rate in CKD with the consequent loss of distensibility and increasing vascular stiffening. The pathogenesis of arterial calcification is an active, tightly regulated cell-mediated process that is subject to regulation [2]. In the early stages, persistent inflammatory stimuli promote microcalcification with osteogenic activity through phenotypic conversion of vascular smooth muscle cells (VSMCs) into osteoblast-like cells. Upregulation of Runx2 in VSMCs orchestrates osteoblastic differentiation by regulating downstream bone-related proteins such as alkaline phosphatase, osteopontin and osteocalcin [3, 4]. Altered regulation of matrix metalloproteinases (MMPs) has also been shown to predispose to extracellular matrix degradation and facilitate arterial calcification [5]. Release of matrix vesicles and apoptotic bodies from dying VSMCs has been shown to be another important mechanism involved in vascular calcification [6].

Vitamin D is one of the most important steroid hormones in the human body. Signaling components of the vitamin D hormonal system are widely expressed across multiple cell types of the cardiovascular system, including VSMCs and this reflects its putative role in the regulation of cardiovascular health [7, 8]. In CKD, perturbation of the vitamin D hormonal system has been associated with the development of cardiovascular [9, 10], bone-mineral [11] and renal [12] complications, contributing directly to premature death [9, 10]. In fact, low concentrations of 25-hydroxyvitamin D (25-OH-D) are associated with an increased risk of cardiovascular mortality [13]. As CKD progresses, activity of 1α-hydroxylase (1α-OHase or CYP27B1) in the kidney, the major enzyme responsible for synthesizing active 1,25-dihydroxyvitamin D (1,25-OH-D) decreases [14]. This has led to the widespread use of the endogenous hormone, calcitriol and analogues, such as Paricalcitol (19-nor-1α,25(OH)_2_D_2_) to treat CKD-associated secondary hyperparathyroidism. These agents effectively suppress parathyroid hormone (PTH) as part of the endocrine regulatory system. Paricalcitol allosterically activates the vitamin D receptor (VDR) and has been reported to have altered calcaemic properties [15]. Increasing evidence suggests that human arteries are not only responsive to endocrine 1,25-OH-D and analogues by expressing VDR, but are also sites for active local vitamin D tissue synthesis [7, 16].

The role of vitamin D, its derivatives and signaling system in vascular calcification is complex. VDR knockout mice develop significant vascular calcification, as well as upregulation of the renin-angiotensin-aldosterone (RAAS) system, hypertension, left ventricular hypertrophy and heart failure [17–19]. This body of evidence has provided rationale for therapeutic interference by VDR agonist (VDRa) therapy. However, VDRa therapy in animal models has yielded apparent disparate results. Administration of pharmacological doses of calcitriol resulted in increased aortic calcification in rats with CKD, however this was not seen in animals treated with synthetic paricalcitol [15]. In another model, both calcitriol and paricalcitol were protective against vascular calcification at dosages sufficient to correct secondary hyperparathyroidism [20]. Additionally, cardiovascular outcomes trials have also yielded conflicting results [21–24].

Discrepant cardiovascular outcome data may be attributable to several factors such as choice of intervention, population characteristics and sample size, and the impact of impaired kidney function and the uremic milieu on vitamin D metabolism. This is further compounded by physiological differences between animal models and humans, and inherent limitations of species differences and single-cell *in vitro* preparations to represent true *in vivo* responses in humans [8]. Additionally, of critical significance is that alterations in the vitamin D signaling system, metabolism and biological functions of VDRa in the human arterial system in health and CKD is still incompletely understood. In this study, we therefore sought to address this by conducting the first comprehensive analysis of alterations of the vitamin D signaling system (including phenotypic and enzymatic changes) in human arteries from healthy and CKD patients and under calcifying conditions. Additionally, we assessed how these arterial changes influence their exposure to VDRa, the mechanisms involved by evaluating critical regulators of arterial calcification and their potential therapeutic properties. Given that calcitriol and its analogue, Paricalcitol may have varying calcemic properties, we evaluated the effects of both these VDRas using arterial organ cultures, *in vivo*. The present study provides critical data on complex alterations of arterial vitamin D signaling components and metabolic pathways that occur in CKD which will help inform future interventional studies.

## Methods

### Human arterial explant culture

Human renal and epigastric artery collection was performed during kidney transplantation and elective nephrectomy respectively at the University Hospital Coventry and Warwickshire NHS Trust after obtaining written informed consent. Arteries were collected between 12 January 2012 to 13 March 2013. Fresh surgically removed arteries from 12 healthy kidney donors (control) and 12 patients with end-stage CKD undergoing renal transplant (CKD) (Table 1) were cut into small rings (approx. 2 mm in length and 2–3 mm in diameter). They were equilibrated and washed for 1hour in plain VSMC growth medium. Arterial explants were cultured in VSMC growth medium 2 supplemented with 0.5% BSA for 14 days and treated with calcitriol (100nM) or paricalcitol (300nM) in normal (1.1mmol/L Ca^2+^) or calcifying (5mmol/L Ca^2+^) conditions. 5mmol/L β-glycerophosphate (β-GP) was added to facilitate mineralization. Following treatments arterial rings were washed and snap frozen in liquid nitrogen. The tissues were homogenized in liquid nitrogen and resulting lysate was clarified by microcentrifugation at 10,000g for 10 min at 4°C for further protein or RNA analysis.

**Table 1:** Clinical characteristics of arterial donors for organ cultures.

Variables	CKD	Control	p–value
**No. of donors**	12	12	-
**Age, years**	47.8	55.7	0.17
**Male, n (%)**	7(58)	4(33)	0.23
**Ethnicity**			
**caucasian / Asian/black**	8/2/2	11/1/0	0.25
**BMI, kg/m**^**2**^	26.3±3.1	23.6±3.4	0.05
**Smoking ever, n (%)**	6 (50)	2(29)	0.15
**Hypertension, n (%)**	11(92)	0(0)	-
**Systolic BP, mm Hg**	135±17.2	120.3±8.0	0.04
**Diastolic BP, mm Hg**	80±9.8	69.9±14.3	0.08
**Diabetes mellitus, n (%)**	1(8)	1(8)	0.15
**Dialysis, n (%)**			
**Predialysis**	2(17)	-	-
**CAPD**	3(25)	-	-
**Hemodialysis**	7(58)	-	-
**Dialysis vintage, months**	34 (0–90)	-	-
**Creatinine, mg/dl**	7.0±2.5	0.19±0.96	<0.001
**eGFR, ml/min/1.73m**^**2**^	8.8±3.6	77.4±14.6	<0.001
**Hemoglobin, g/dl**	11.9±1.2	12.9±1.0	0.05
**Calcium, mg/dl**	9.1±0.3	8.9±0.56	0.48
**Phosphate, mg/dl**	6.0±1.4	4.2±0.88	<0.01
**PTH, pg/ml**	53.5±58.6	3.4±1.6	0.02

Data are mean ± SD, median (Range) or frequencies (%). BMI, body mass index; BP, blood pressure; eGFR, estimated glomerular filtration rate; CKF, patients with chronic renal failure; Control, donors with maintained renal function. P–value by paired-samples t-test.

### Cell culture

Primary cultures of HA-SMCs (PromoCell) were maintained in SMC growth medium 2 containing 5% foetal calf serum (FCS), 0.5 ng/ml epidermal growth factor, 2.0 ng/ml basic fibroblast growth factor and 5μg/ml insulin (PromoCell). The cells were grown in 5% CO2 at 37°C in medium renewed every 3 days. Confluent cells were detached by trypsin/EDTA and subcultured with a split ratio 1:2. HAoSMC were used between 2 and 5 passages.

### Antibodies and assays

Cell and tissue lysates were separated by SDS-PAGE and Western blotted with anti-human VDR (C-20) rabbit polyclonal antibody (Santa Cruz Biotechnology sc-1008), anti-mouse 1α-OHase (CYP27B1, sigma, M8642) and anti-human 24-OHase (CYP24A1, mouse monoclonal, sigma, WH0001591M7) mouse monoclonal antibody. HCK-8 cells were used as a positive control. Tissue lysates were assayed for VDR, CYP27B1, CYP24A1 and Runx2 using human VDR ELISA Kit (MBS160161, MyBioSource), human 1α-OHase (CYP27B1) ELISA kit (MBS937445, MyBioSource), human 24-OHase (CYP24A1) (MBS811442, MyBioSource) ELISA kit and human RUNX2 ELISA kit (MBS027654, MyBioSource) following the manufacturer’s protocol. VDR, CYP27B1, CYP24A1 and Runx2 expression was normalized against protein concentration of the samples. Analysis of arterial calcification was performed using the orthocresolphthalein complexone method. 1,25-OH-D levels were assessed using an enzyme immunoassay (EIA) (AC-62F1, IDS). Detailed assay protocols are provided in the supplemental methods.

### Data analysis

Continuous variables were summarized by using means (standard deviations, SD) when normally distributed and by medians (interquartile ranges [IQRs]) otherwise. Categorical variables are presented as frequencies with percentages. Two group comparisons between groups were conducted by two-tailed paired t-test after testing for normality. One-way ANOVA followed by Tukey’s multiple comparison test was used for comparing means of more than two groups. The statistical analysis was conducted in STATA (version 14) software, and P < 0.05 was regarded as statistically significant.

### Study oversight

Ethical approval for this study was obtained from Coventry Research Ethics Committee (05/Q2802/26), UK. None of the transplant donors were from a vulnerable population and all donors or next of kin provided written informed consent that was freely given.

### Supplemental methods

Detailed description for immunohistochemistry, Western blotting, and polymerase chain reaction protocols used is provided in the supplemental methods section.

## Results

### Expression of vitamin D signaling elements is altered in CKD arteries

**Fig 1:**
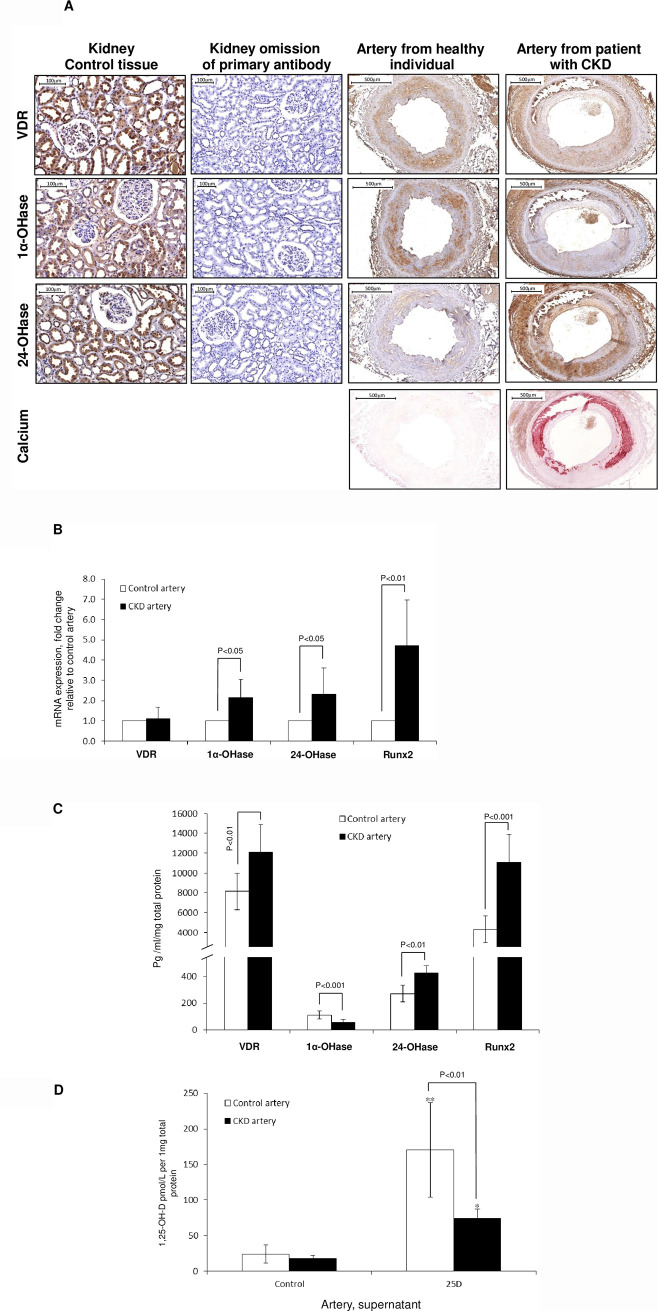
The vitamin D signalling system in human arteries from healthy and CKD patients. **A)** The VDR and 1α-OHase is expressed in arteries from healthy donors, with strong expression across the medial smooth muscle layer and endothelium. CKD arteries exhibited suppressed VDR and 1α-OHase expression. 24-OHase expression is high in arteries from CKD patients across the medial smooth muscle layer and endothelium but suppressed in healthy arteries. CKD arteries exhibited significant medial calcification; **B)** RNA and **C)** protein expression of VDR, 1α-OHase and 24-OHase expression in arterial lysates; **D)** 1,25-OH-D production is in arteries following 25-OH-D treatment, but production is blunted in CKD arteries. *p<0.05, **p<0.01.

We first sought to characterize the expression of vitamin D signaling components in human arteries from CKD and control patients. Baseline data of donor patients are summarised in Table 1. Immunohistochemical staining of arterial sections (Fig 1A) revealed the presence of VDR, 1α-OHase and 24-OHase in human arteries.

We identified upregulated basal mRNA (p<0.001) expression of 1α-OHase (p<0.01), but suppressed protein expression (p<0.001) in CKD arteries compared to control (Fig 1A and 1B). Both 24-OHase (p<0.05) mRNA and protein (p<0.01) expression were upregulated in CKD arteries compared to control (Fig 1B and 1C). While VDR mRNA expression appeared unchanged in CKD arteries compared to control, basal protein expression was significantly upregulated in CKD arteries (p<0.01). mRNA expression of Runx2, a critical regulator of osteogenic transformation, was dramatically (almost 5-fold, p<0.01) increased in CKD artery explants (Fig 1B), as was protein expression (p<0.01, Fig 1C).

We next compared the ability of CKD versus control arterial explants in culture to synthesise 1,25-OH-D. Whereas 1,25-OH-D production was clearly demonstrated in control arterial explants (7-fold, p<0.05, Fig 1D) and suggests 1α-OHase activity, 1,25-OH-D production in CKD explants was markedly reduced compared to control arteries, suggesting a reduction in 1α-OHase or increased 24-OHase enzyme activity (p<0.01).

### HA-SMCs express all components of the vitamin D signaling system

Given that vascular smooth muscle cells form the predominant cell type of the arterial wall, we also assessed the vitamin D signaling system in HA-SMCs. Both mRNA and protein expression of VDR, 1α-OHase and 24-OHase were expressed in HA-SMCs (S1A and S1B Fig in S1 Raw images). HKC-8 cells and cortical region of healthy human kidney tissue have been shown previously to express 1α-OHase [25, 26] and were used as positive controls. We found that 1,25-OH-D production was dramatically (17-fold, p<0.001, S1C Fig in S1 Raw images) increased in HA-SMCs cultured with 25-OH-D, indicating 1α-OHase activity.

### VDR modulation by calcitriol and paricalcitol is blunted in CKD arteries

We next sought to determine the role of VDR therapy in regulating the vitamin D signaling system. Because calcitriol and its analogue, paricalcitol (19-nor-1α,25(OH)_2_D_2_) have been reported to have different calcemic properties, we compared their differences using arterial organ culture explants, *in vivo*. Arteries from control and CKD patients were cultured in either normal or calcifying medium and treated with either calcitriol and paricalcitol. In summary, we found that arterial explants from CKD patients exhibit a reduced capacity to synthesise 1,25(OH)2D, increased induction of the vitamin D catabolic pathway in response to VDRa, and respond to calcitriol and paricalcitol effects differentially. These complex changes are summarized in Tables 2 and 3.

**Table 2:** Summary of the vitamin D signalling system, enzymatic and phenotypic alterations in healthy and CKD arteries.

**CONTROL ARTERY PROTEIN EXPRESSION**		**VDR**	**1Α-OHASE**	**24-OHASE**	**RUNX2**	**CALCIFICATION**
Normal medium	Control	**+**	**+**	**+**	**+**	**-**
	Calcitriol	**++**	**+**	**++**	**+**	**-**
	Paricalcitol	**+**	**+**	**++**	**+**	**-**
Calcifying medium	Control	**+**	**+**	**+**	**++**	**+++**
	Calcitriol	**++**	**+**	**++**	**++**	**+++**
	Paricalcitol	**+**	**+**	**++**	**+**	**+++**
**CKD Artery Protein expression**		**VDR**	**1α-OHase**	**24-OHase**	**Runx2**	**Calcification**
Normal medium	Control	**++**	**(+)**	**++**	**+++**	**++**
	Calcitriol	**++**	**(+)**	**++**	**+++**	**++**
	Paricalcitol	**++**	**(+)**	**+++**	**++**	**++**
Calcifying medium	Control	**++**	**(+)**	**+++**	**++++**	**++++**
	Calcitriol	**++**	**(-)**	**+++**	**++++**	**++++**
	Paricalcitol	**++**	**(+)**	**+++**	**++**	**++++**

The table illustrates protein expression changes of the vitamin D signalling system and phenotypic alterations following VDRa treatment. Key:—no changes; (+) little increase; + mild increase; ++ moderate increase; +++ significant increase.

**Table 3:** Summary of osteoblastic transformation, calcium deposition, inflammatory response in CKD and healthy arteries.

**CONTROL ARTERY RNA EXPRESSION**		**CALCIUM**	**RUNX2**	**MMP2**	**MMP9**	**OSTEOC**	**IL6**	**IL10**
Normal medium	Control	**-**	**+**	**+**	**+**	**+**	**+**	**+**
	Calcitriol	**-**	**+**	**+**	**+**	**+**	**+**	**++**
	Paricalcitol	**-**	**(+)**	**+**	**+**	**+**	**(+)**	**++**
Calcifying medium	Control	**++**	**+**	**++**	**++**	**++**	**+**	**++**
	Calcitriol	**++**	**+**	**++**	**+**	**+**	**+**	**++**
	Paricalcitol	**++**	**(+)**	**+**	**+**	**+**	**+**	**+++**
**CKD Artery RNA expression**		**Calcium**	**Runx2**	**MMP2**	**MMP9**	**Osteoc**	**IL6**	**IL10**
Normal medium	Control	**++**	**+++**	**++**	**++**	**+**	**++**	**+**
	Calcitriol	**++**	**+++**	**++**	**++**	**++**	**++**	**+**
	Paricalcitol	**++**	**++**	**+**	**++**	**++**	**++**	**+**
Calcifying medium	Control	**++++**	**+++**	**+++**	**+++**	**+**	**+**	**++**
	Calcitriol	**++++**	**+++**	**+++**	**++**	**++**	**+**	**++**
	Paricalcitol	**++++**	**++**	**++**	**++**	**++**	**+**	**++**

The table illustrates the amount of calcium deposition (calcium) and downstream mRNA changes following VDRa treatment. Key:—no changes; (+) little increase; + mild increase; ++ moderate increase; +++ significant increase.

**Fig 2:**
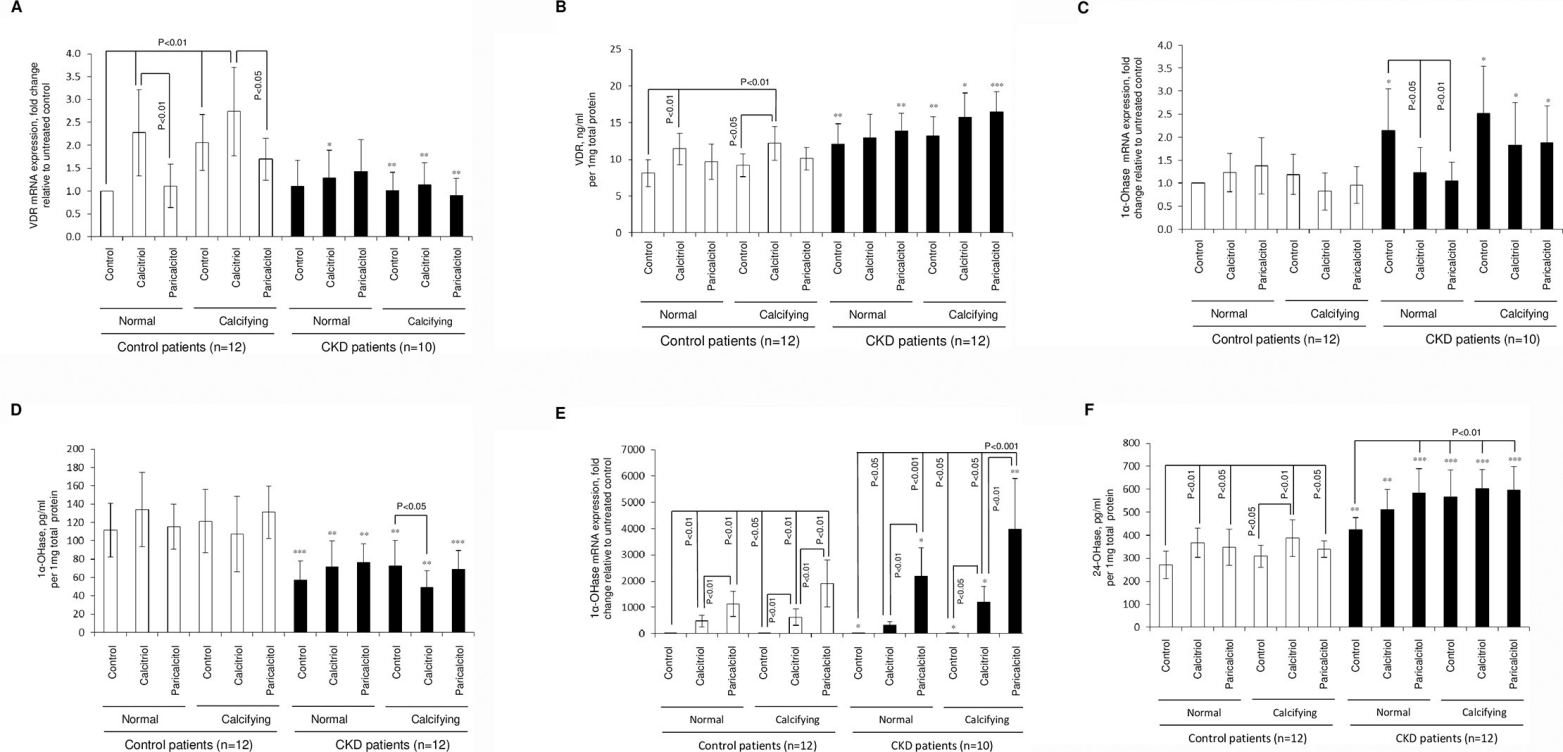
Modulation of the vitamin D signalling system in human arteries following calcifying stress and VDRa therapy. Arterial rings were treated for 14 days *with* calcitriol (100nM) or paricalcitol (300nM) in normal or calcifying medium. **A)** mRNA and **B**) protein for vitamin D receptor (VDR); **C**) mRNA and **D**) protein for 1α-OHase (CYP27B1); **E**) mRNA and **F**) protein for 24-OHase (CYP24A1). * refers to comparison within treatment group between control and CKD artery; *p<0.05, **p<0.01, ***p<0.001.

We observed a considerable increase in VDR mRNA expression (p<0.01, Fig 2A) and protein expression (p<0.01, Fig 2B) in calcitriol-treated control patient explants, but no change in gene or protein expression occurred after treatment with paricalcitol. VDR mRNA levels were significantly up-regulated in calcifying cultures compared to normal medium cultures (p<0.01). Interestingly, in CKD patients basal VDR mRNA expression was similar to control arteries, while there were no significant changes in VDR mRNA levels following incubation in calcifying medium compared to control, or treatment with calcitriol or paricalcitol (p<0.05 to p<0.01). In CKD arteries, VDR protein levels were moderately elevated (p<0.05 to p<0.001) compared to respective control explants, with no response observed after calcitriol or paricalcitol stimulation even when exposed to calcaemic conditions (Fig 2B). These changes suggest raised VDR expression occurs in CKD arteries compared to arteries from patients with maintained, normal renal function. The raised expression of VDR did not translate into a VDRa response as observed for calcitriol in control arteries.

### Calcitriol and paricalcitol suppress arterial 1α-OHase and stimulate 24-OHase expression in CKD arteries

1α-OHase mRNA and protein expression did not change after exposure to VDRa’s and / or calcifying conditions in control artery (Fig 2C and 2D). Compared to control arteries, increased 1α-OHase mRNA expression in CKD arteries under both normal (p<0.05) and calcifying conditions (p<0.05) was only significantly suppressed by VDRa under normal conditions (calcitriol, p<0.05; paricalcitol, p<0.01, Fig 2C). In contrast 1α-OHase protein expression for all *in vitro* treatment settings remained significantly reduced when compared to control artery (p<0.01 to p<0.001). And in contrast to the observation for 1α-OHase mRNA suppression with VDRa in CKD artery under normal conditions, reduced 1α-OHase protein expression was only observed for calcitriol under calcifying conditions (p<0.05), Fig 2D). Vitamin activation in CKD arteries appears to remain suppressed also under calcifying condition.

As expected, incubation with calcitriol or paricalcitol resulted in a marked (p<0.05 to p<0.01, Fig 2E) up-regulation of 24-OHase mRNA expression in control and CKD artery explants under basal or calcifying conditions. Analysis of 24-OHase protein expression revealed similar but less dramatic responses, with increased 24-OHase protein expression after treatment with calcitriol or paricalcitol in control artery (p<0.05 to p<0.01). This response was blunted under calcifying conditions when arteries were treated with paricalcitol. In CKD arteries, the already increased expression of 24-OHase protein under normal conditions was further increased when arteries were exposed to VDRa, particularly paricalcitol (p<0.01) or exposed to calcifying medium (p<0.01). Exposure of CKD arteries to VDRa treatment in the context of calcifying conditions did not increase the already stimulated 24-OHase protein expression further (Fig 2F). 24-OHase responds in control arteries to VDRa with increased expression and activity. The response is as expected for vitamin D target tissue and allows hormonal regulation of the tissue. In CKD arteries the increased 24-OHase expression under normal condition, after VDRa exposure and also under calcifying conditions results with a tissue deprived VDR activation. These data are summarised in Table 2.

### Differential modulation of major regulators of arterial calcification by calcitriol and paricalcitol

**Fig 3:**
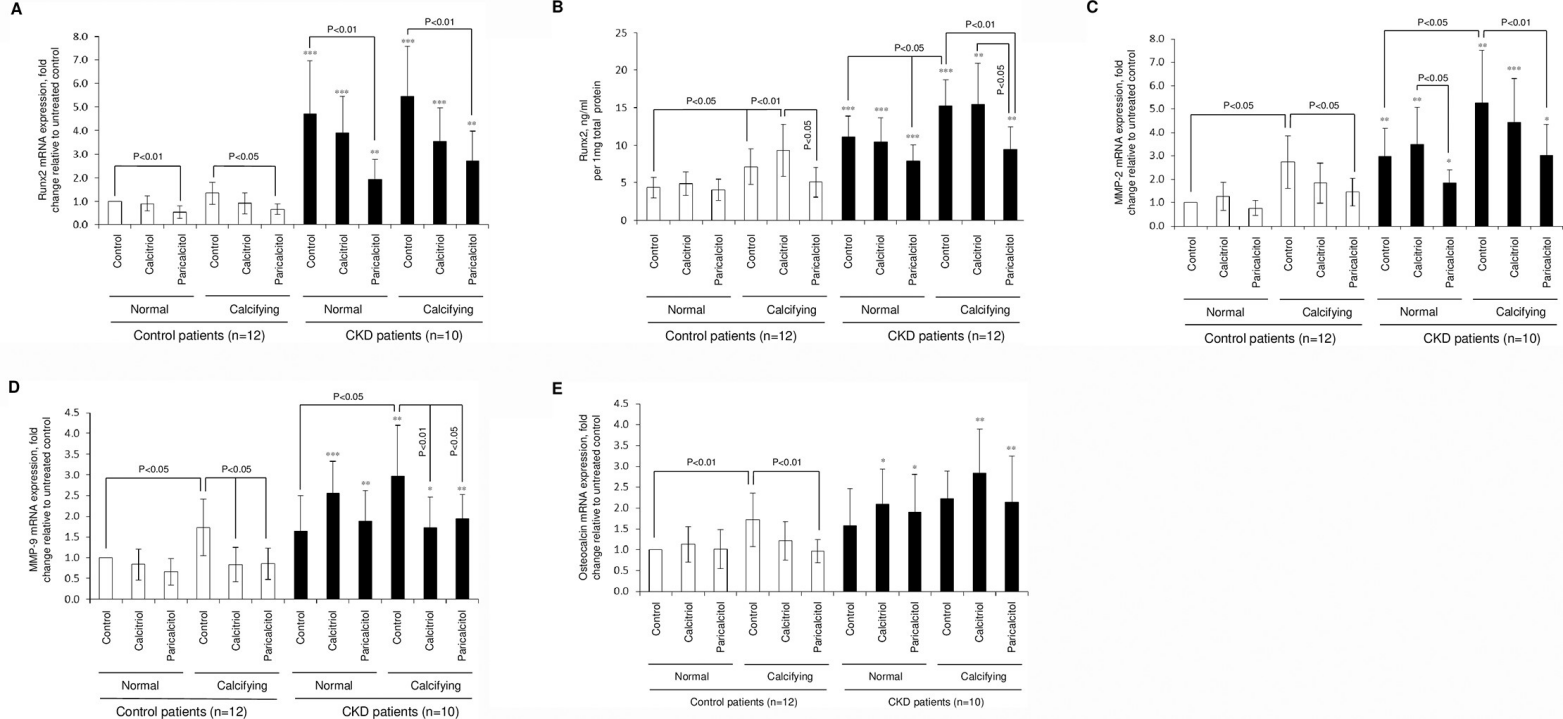
Modulation of arterial phenotype following calcifying stress and VDR therapy. Arterial rings were treated for 14 days *with* calcitriol (100nM) or paricalcitol (300nM) in normal or calcifying medium. **A)** mRNA and **B**) protein for Runx2; **C)** mRNA quantification for MMP-2; **D**) mRNA quantification for MMP-9; **E)** mRNA of osteocalcin. * refers to comparison within treatment group between control and CKD artery; *p<0.05, **p<0.01, ***p<0.001.

Basal arterial mRNA expression of Runx2 in CKD arteries was markedly (up to 5.5-fold) higher compared to the control group (p<0.01 to p<0.001, Fig 3A). In control explants, mRNA Runx2 expression was markedly down-regulated (p<0.05 to p<0.01) in paricalcitol-treated cultures under basal (p<0.01) and calcifying (p<0.05) conditions, but did not change significantly after calcitriol treatment. Following paricalcitol treatment of CKD explants, the increased basal Runx2 mRNA (p<0.001 compared to control artery) level was partially reduced (p<0.01), but still significantly higher than in respective control cultures (p<0.01). The same effect was observed under normal or calcifying conditions. Changes in Runx2 protein expression generally mirrored Runx2 mRNA patterns. Runx2 protein expression in normal and treatment groups were much higher in CKD arteries than in control arteries when comparing the corresponding treatment groups (<0.01–0.001). Both artery groups, control (p<0.05) and CKD artery (p<0.05) responded to high calcium and phosphate exposure in culture medium with a significand increase of Runx2 protein expression, Fig 3B). While, osteoblastic artery smooth muscle transformation with increased Runx2 expression is known to occur in arteries exposed to uremic conditions, the data here indicates that high calcium and phosphate conditions seems drive this phenotype as observed not only for CKD, but also for control artery. Only paricalcitol was found to have an inhibitory effect on artery cell phenotype transformation.

We next assessed the expression of matrix metalloproteinases (MMP)-2 and -9, both critical regulators of phenotypic VSMC conversion and matrix remodelling. In CKD explants basal and agonist-treated MMP-2 mRNA levels were considerably higher than in respective control cultures (p<0.05 to p<0.001, Fig 3C). Paricalcitol treatment effectively inhibited calcium and phosphate induced up-regulation of MMP-2 mRNA in both control (p<0.05) and CKD arteries (p<0.01), while calcitriol treatment did not produce significant changes in MMP-2 gene expression. Changes in MMP-9 mRNA expression exhibited a similar pattern to MMP-2 mRNA expression. MMP-9 mRNA expression in CKD arteries was markedly higher than in control group (p<0.05 to p<0.001, Fig 3D). We also observed a pronounced increase in MMP-9 levels under calcifying culture conditions for control (<0.05) and CKD artery (<0.05), which was suppressed following treatment with both, calcitriol or paricalcitol (p<0.05 to p<0.01). Another downstream bone-derived marker, osteocalcin, exhibited a similar expression pattern. Osteocalcin mRNA was upregulated in control arteries treated in calcifying conditions (p<0.01) but remained suppressed with paricalcitol treatment (p<0.01, Fig 3E). Osteocalcin mRNA in all CKD artery treatment groups was up-regulated and interestingly, high calcium conditions or VDRa treatment did not change the expression.

### Modulation of inflammatory markers by calcitriol and paricalcitol

**Fig 4:**
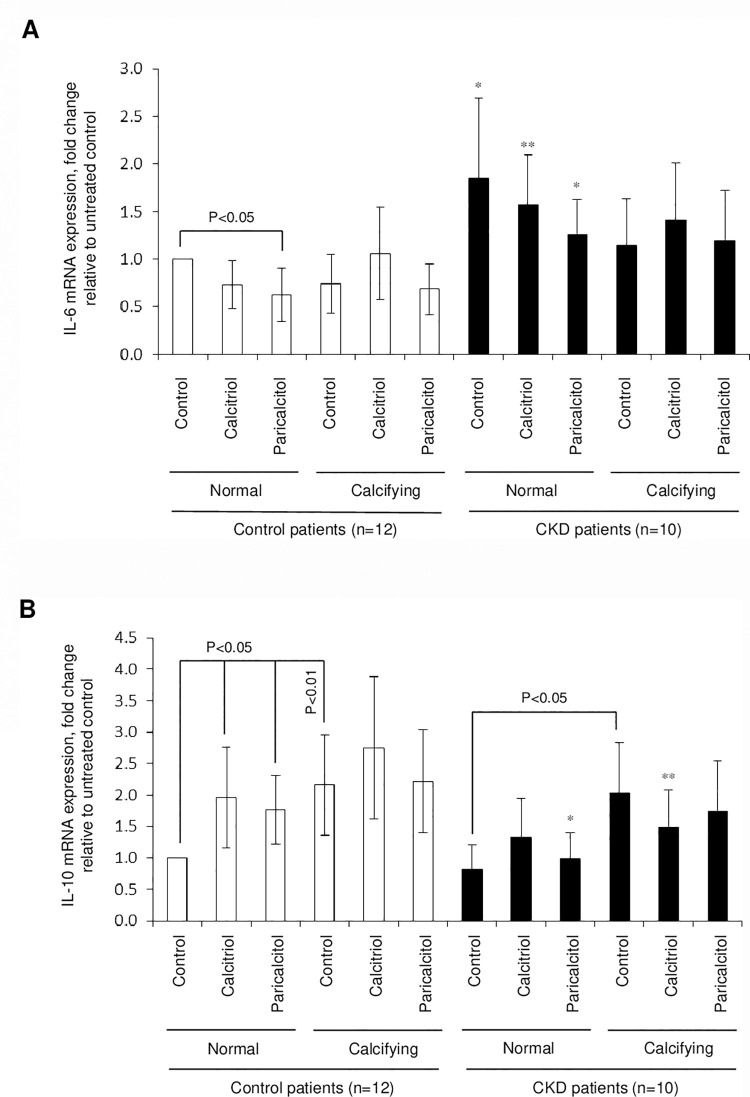
Treatment of arteries with calcifying stress and VDR activators alters vascular inflammatory molecule synthesis. Arterial rings were treated for 14 days *with* calcitriol (100nM) or paricalcitol (300nM) in normal or calcifying medium. **A)** IL-6 mRNA quantification; **B)** IL-10 mRNA quantification. * refers to comparison within treatment group between control and CKD artery; *p<0.05, **p<0.01.

IL-6 mRNA expression in CKD arteries was higher compared to the control arteries only when not exposed to high calcium and phosphate concentrations (p<0.05 to <0.01, Fig 4A). In control explants, IL-6 expression was markedly down-regulated (p<0.05) in paricalcitol-treated artery cultures but not after calcitriol treatment. In contrast, CKD artery explant IL-6 mRNA expression was not reduced after calcitriol or paricalcitol treatment, irrespective of normal or exposure to calcifying conditions. Exposure to calcifying medium resulted in increased IL-10 mRNA expression in both, control (p<0.01) and CKD arteries (p<0.05). IL-10 mRNA levels however, were increased after calcitriol and paricalcitol treatment in control explants (p<0.05, Fig 4B), but unchanged in CKD explants. These data was summarised in Table 3.

### VDRa exposure does not modulate calcification in arterial explants

**Fig 5:**
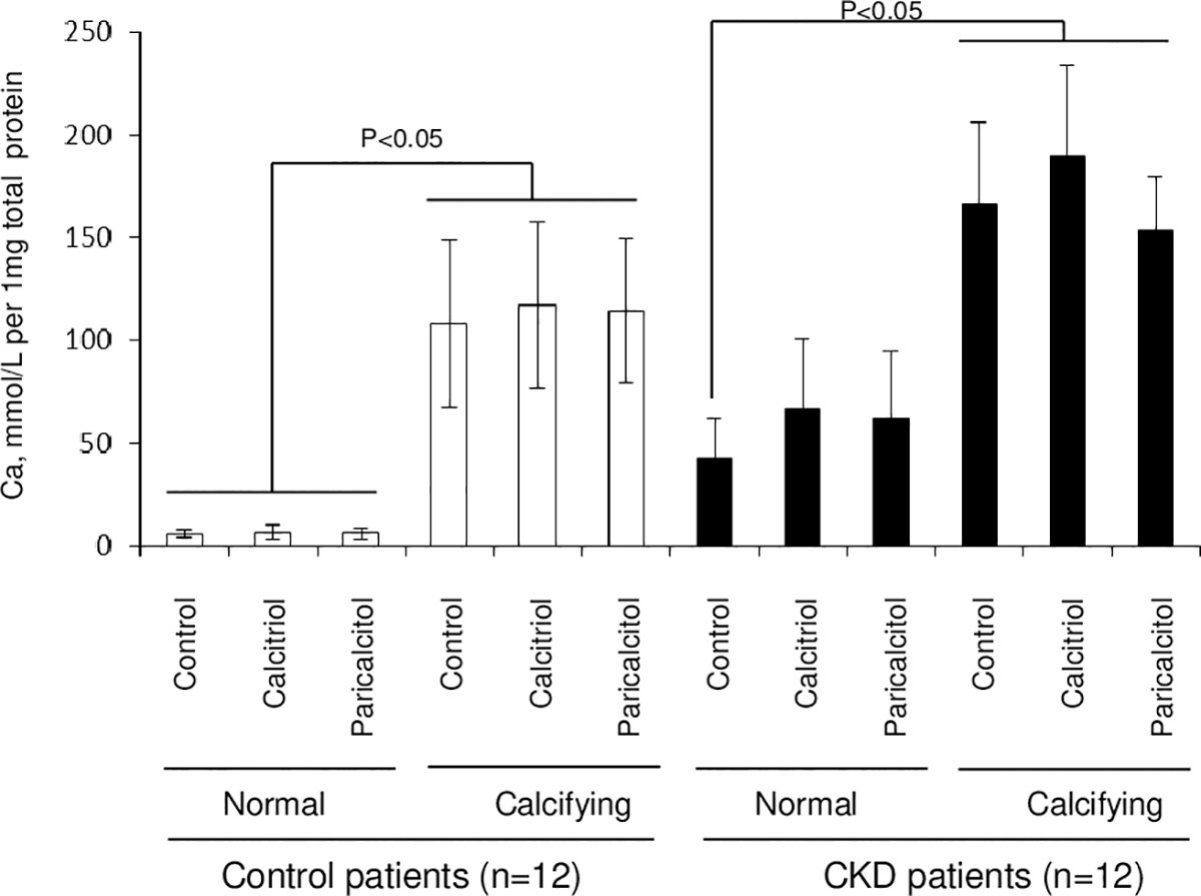
Treatment of arteries with VDR activators does not alter arterial calcium content. Arterial rings were treated for 14 days *with* calcitriol (100nM) or paricalcitol (300nM) in normal or calcifying medium. Arteries were assessed for calcification using the orthocresolphthalein complexone method. Arteries cultured in calcifying medium exhibited increased calcium content. Treatment of arteries with either calcitriol or paricalcitol did not altered calcium content.

Incubation of arterial explants from healthy and CKD patients in calcifying medium resulted in a marked up-regulation of calcium deposition in both artery groups (p<0.05; Fig 5). Arterial calcium content was not modulated, increased or reduced with calcitriol or paricalcitol treatment in both, control and CKD arteries. Table 3 summarises the findings of osteoblastic transformation, calcium deposition, inflammatory response with treatment of normal and CKD artery.

Because vascular calcification is widely considered a hallmark of arterial aging, to further explore the role VDRa therapy, we assessed the expression of senescence associated β-galactosidase (SAβG), an established aging markers that accumulates only in senescent cells [27]. In a proof-of-concept experiment, HA-SMCs were treated with both native active vitamin D (calcitriol) and inactive vitamin D (calcidiol) (S2 Fig in S1 Raw images). We found that both calcitriol and calcidiol reduced expression of SAβG significantly in HA-SMCs under calcifying stress.

## Discussion

Vitamin D effects are mediated via the VDR, a member of the steroid receptor superfamily and a nuclear ligand-activated transcription factor forming the transcriptional pre-initiation complex [28]. VDR presence has been demonstrated in more than 30 human tissues, including kidney, bone, intestine, parathyroid glands, immune cells, myocardium and SMC [29]. To become active, vitamin D must undergo hydroxylation in the liver to form 25-OH-D, which is further hydroxylated by 1α-OHase enzyme in the kidney to produce active 1,25-(OH)_2_-D [8]. We provide evidence that normal human arteries express all critical components of the vitamin D system (VDR, 1α-OHase and 24-OHase). However, their arterial alterations in health and CKD, and following exposure to calcifying environments is complex and influences the response to VDRA exposure as summarized in Tables 2 and 3.

Immunohistochemical analysis of VDR, 1α-OHase and 24-OHase showed strong staining in the medial layer of the artery, with visible staining of SMCs under high magnification. Consistent with previous findings in kidney [26], staining for 1α-OHase and 24-OHase in VSMC was cytoplasmic. The VDR appeared to be present both in the cytoplasm and nuclei. Furthermore, VDR, 1α-OHase and 24-OHase staining was also present in the endothelium. The presence of 1α-OHase and VDR protein and synthesis of 1,25(OH)_2_D has been described previously in human vascular endothelial cells [30]. Comparative analysis of basal vitamin D system component expression in human arteries revealed a significant increase in VDR and 24-OHase levels and a reduction of 1α-OHase in CKD arteries. Importantly, this was accompanied by a marked up-regulation of Runx2, a key osteoblastic transcription factor, in CKD explants. Impaired vascular production of active vitamin D and its increased degradation by 24-OHase may exacerbate circulating 1,25-OH-D deficiency observed in CKD patients in the context of 25-OH-D deficiency, which has been shown to be closely associated with cardiovascular morbidity and mortality [31].

Further analysis revealed increased levels of VDR protein in CKD explants, both in basal and agonist-treated cultures. Interestingly, calcitriol treatment increased VDR expression in control but not in CKD arteries, while paricalcitol induced no significant changes. Finch et al. (2001) previously found that calcitriol and paricalcitol had similar potency in up-regulating VDR content in human osteoblast-like cell line MG63 [32], while Becker et al. (2011) demonstrated a significant elevation of VDR expression in the aorta of unnephrectomized ApoE knockout mice following paricalcitol or calcitriol treatment [33]. The absence of response to VDR activators in CKD explants observed in our study may reflect an impaired adaptive mechanism regulating VDR stimulation.

1α-OHase and 24-OHase are key enzymes regulating metabolism of vitamin D [34]. Our analysis of arterial explants revealed significantly lower expression of 1α-OHase protein in CKD explants, irrespective of treatment with calcitriol, paricalcitol or high calcium and phosphate treatment when compared to control arteries. Measurement of 1,25-dihydoxyvitamin D production confirmed a pronounced decline in 1α-OHase activity in analysed CKD arteries compared to control explants or HA-SMCs. Importantly, while it is well known that the renal 1α-OHase is progressively lost during CKD stages 3–5, there is no evidence that extra-renal 1α-OHase is affected at any stage of the disease [35]. Consequently, our data point to a decrease in the extra-renal (arterial) activity of 1α-OHase in CKD arteries. Extra-renal 1α-OHase has been thought to play an important physiological role by augmenting circulating 1,25-OH-D with its local production [36, 37]. Local production of active vitamin D is crucial for “nonclassical” regulation of normal physiology. These findings unveil an important autocrine/paracrine role of 1α-OHase in SMCs, initially identified in human endothelium by our group, and further demonstrate impaired vitamin D activation in CKD arteries [7].

24-OHase, responsible for degradation of 1,25-OH-D, plays a key role in calcium and vitamin D homeostasis [34]. Up-regulation of 24-OHase mRNA and protein expression in CKD arteries observed in our experiments was further enhanced by treatment with calcitriol and paricalcitol, suggesting markedly increased catabolism of active vitamin D in CKD arteries. Our findings are supported by recent studies of human bronchial SMC and coronary SMC, which showed a strong induction of 24-OHase mRNA after calcitriol or paricalcitol stimulation and confirmed functionality of VDR in these cells [38]. Ultimately, it is the balance between vitamin D activation by 1α-OHase and its inactivation by 24-OHase that determines how much 1,25-OH-D is present in tissues for VDR binding and stimulation.

Differentiation of vascular SMCs into an osteoblastic phenotype is driven by the up-regulation of key transcription factors, including Runx2, which regulates SMC development and controls the expression of a number of osteogenic proteins, such as osteocalcin, osteonectin, alkaline phosphatase and collagen-1 [6, 39]. We found that Runx2 levels were markedly increased in CKD explants, and effectively reduced following treatment with paricalcitol, while calcitriol failed to inhibit Runx2 levels. Arterial calcium content in CKD explants appeared to be increased, however, it was not significant due to a considerable variation between CKD patients and rather small (12 patients in each group) cohort. Despite a marked reduction in Runx2 levels, calcitriol or paricalcitol failed to significantly inhibit average calcium deposition. This could be due to the relatively short (14days) period of observation or the model of isolated artery used in vitro under static culture condition and culture medium. It is possible that the differences observed in gene and protein expression of vitamin D regulatory pathway components in CKD arteries is driven by osteogenic transformation, with osteocyte-like VSMC unable to respond to vitamin D receptor agonists in the same way as healthy VSMCs.

Matrix metalloproteinases (MMPs) represent a family of zinc-dependent endopeptidases that cleave protein components of extracellular matrix and regulate cell migration. MMP-2 and MMP-9 (type IV collagenases) are involved in the breakdown of type IV collagen, the major structural component of basement membranes. Both MMP-2 and MMP-9 are involved in osteoblastic bone formation and osteoclastic bone resorption. Calcitriol was found to activate MMP-2 in human osteoblast-like cells, which may promote bone formation and stimulate bone resorption [40]. Importantly, MMP-2 and MMP-9 contribute to myocardial remodelling and arterial calcification with MMP-2 being up-regulated and closely associated with phosphatemia in patients with CKD [41]. Moreover, in these patients low levels of calcitriol and increased levels of MMP-9 and phosphate were associated with increased arterial stiffness [42]. A strong up-regulation of MMP-2 and MMP-9 expression in arteries from CKD patients and in calcifying medium cultures observed in our study, was effectively blocked by paricalcitol (MMP-2) or both calcitriol and paricalcitol (MMP-9). Given that increases in MMP-2 and MMP-9 activity may exacerbate arterial stiffening in the setting of vascular inflammation, these data suggest a possible anti-inflammatory and protective role for calcitriol and paricalcitol in muscular artery in the context of CKD.

In summary, we have conducted a comprehensive analysis of the vitamin D signaling system and the role of VDR therapy in regulating arterial health. We have demonstrated that human vascular SMCs and arteries express a functionally active vitamin D system–VDR, 1α-OHase and 24-OHase. Arterial explants from CKD patients exhibit a reduced capacity to synthesise 1,25(OH)2D whilst displaying both increased basal expression and excessive induction of the vitamin D catabolic pathway in response to VDRa. The implication is that in CKD, muscular artery may be exposed to low tissue concentrations of 1,25(OH)2D and may blunt the effects of VDRas. This finding is of particular relevance given the contemporary practice of treating the secondary hyperparathyroidism of CKD with supraphysiological doses of 1α-hydroxylated vitamin D compounds. Our experimental model may overcome this localised deficiency by exposing tissues directly to VDRas. The attenuation of Runx2 expression in CKD artery by exposure to VDRa and the associated inhibition of metalloproteinase expression suggests that restoration of vitamin D receptor engagement may have a role in maintaining arterial health in CKD.

## Supporting information

S1 Raw images (PDF)
