# Supplementary material for: Impaired arterial vitamin D signaling occurs in the development of vascular calcification
Source: PLoS One. 2020 Nov 19;15(11):e0241976. doi: 10.1371/journal.pone.0241976 (PMC7676703; doi:10.1371/journal.pone.0241976)

Supplementary Information

S1 Figure: VDR (Vitamin D Receptor) Western Blot

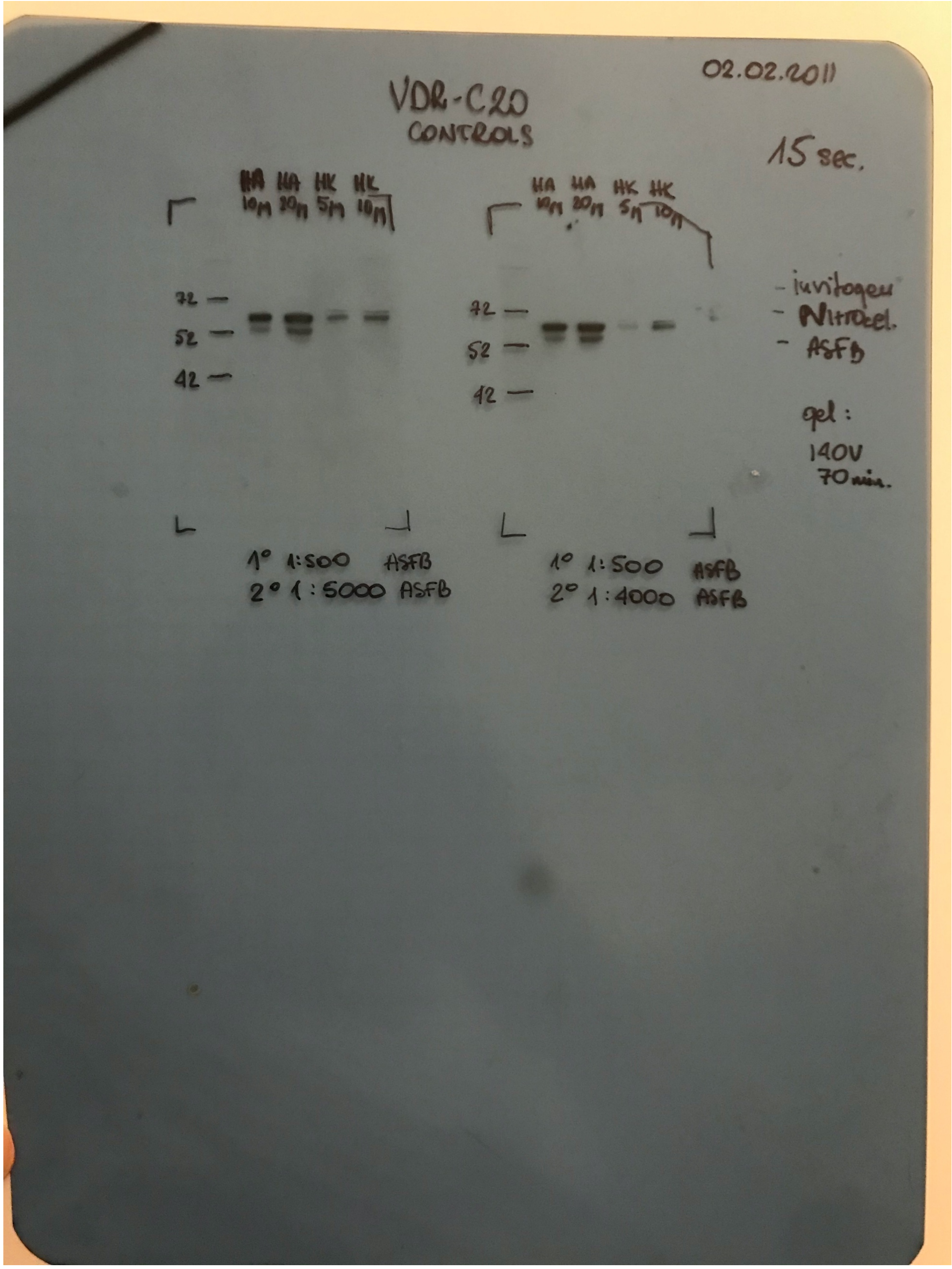

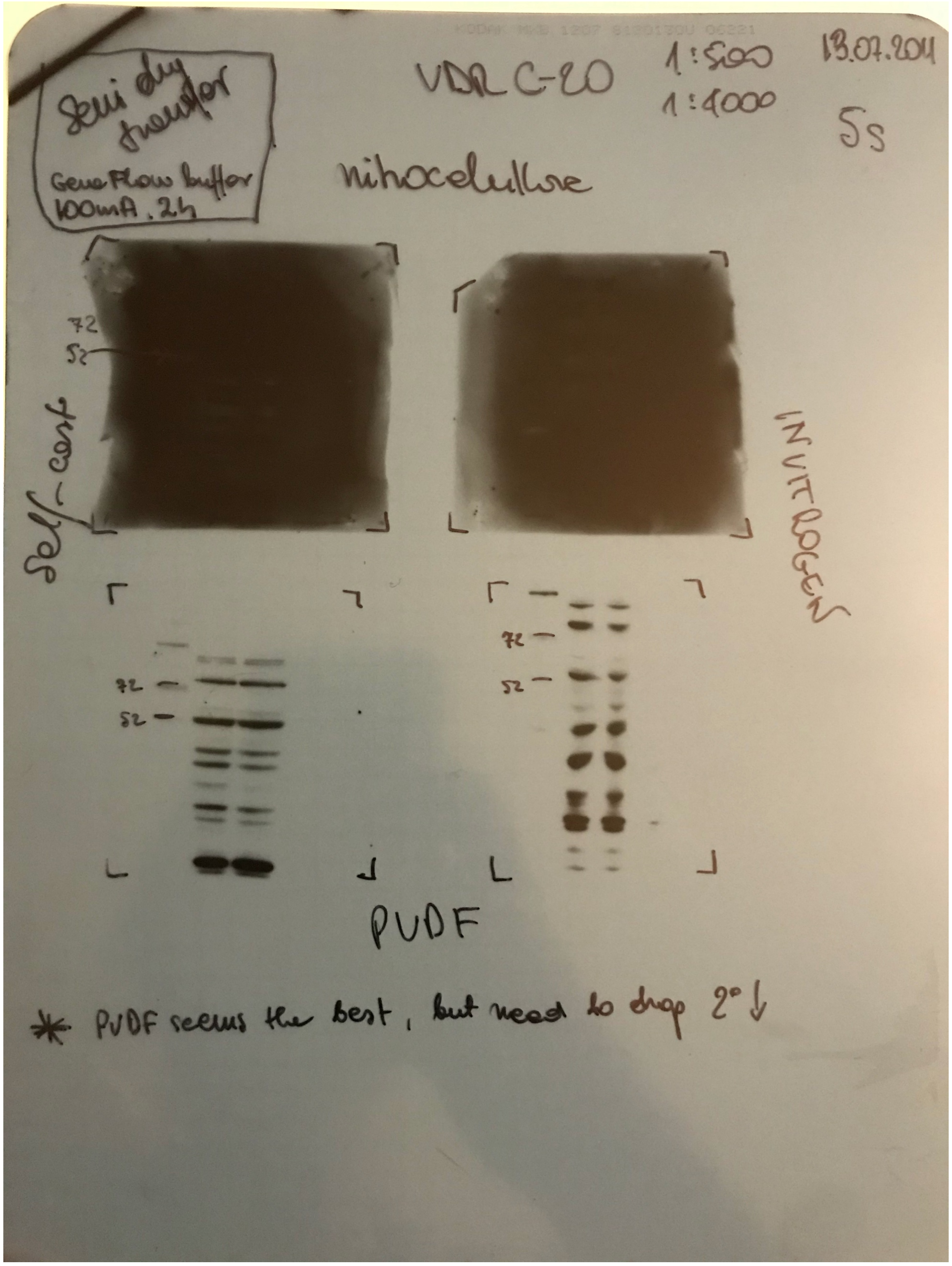
Semi dry transfer
GeneFlow buffer
100mA, 2h
VDR C-20
1:500
1:4000
13.07.2014
5s
nitrocelullose
72
52
self-cast
INVITROGEN
72
52
72
52
PVDF
* PVDF seems the best, but need to drop 2° ↓

S2 Figure: 1α-OHase Western Blot

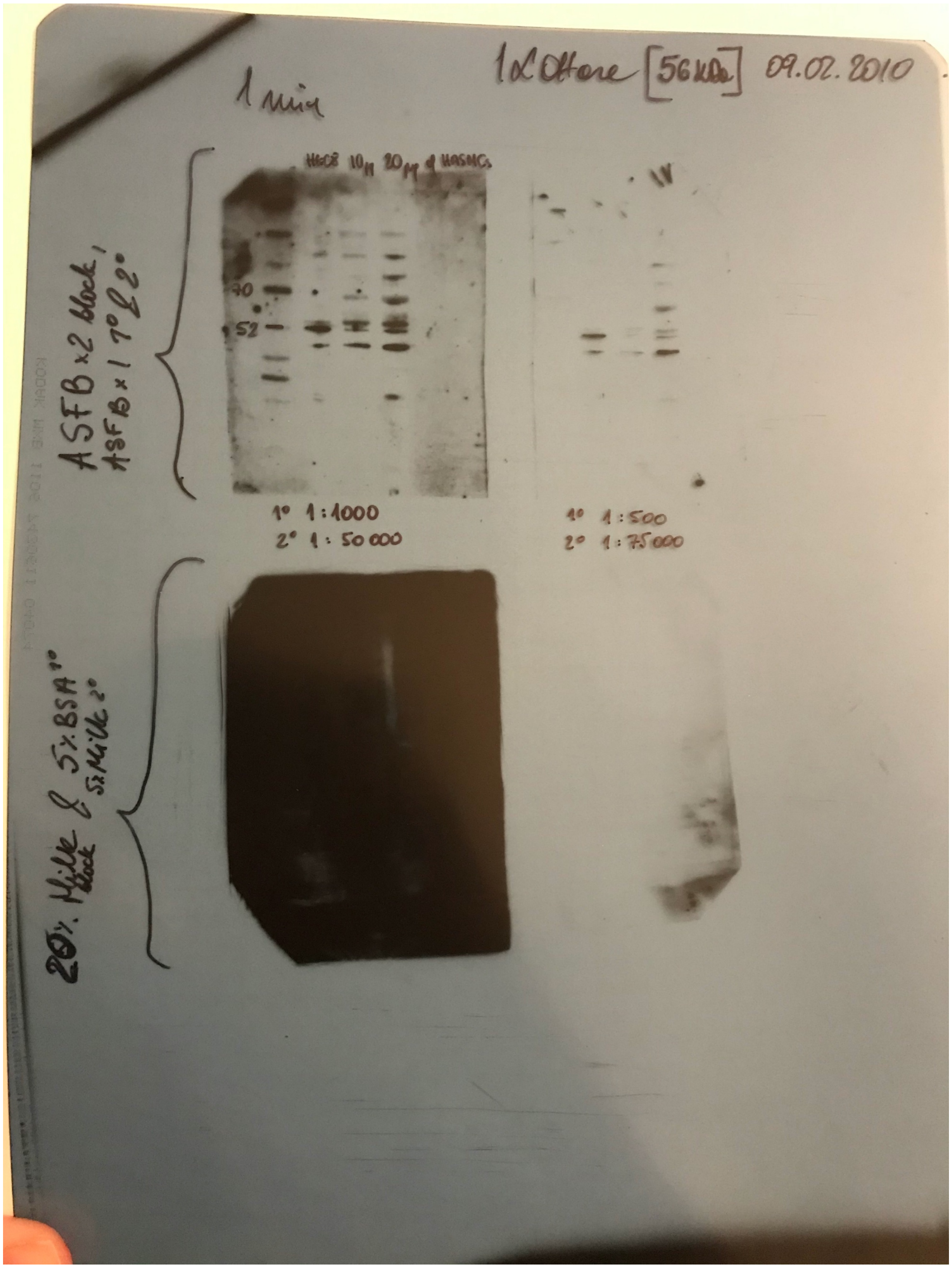

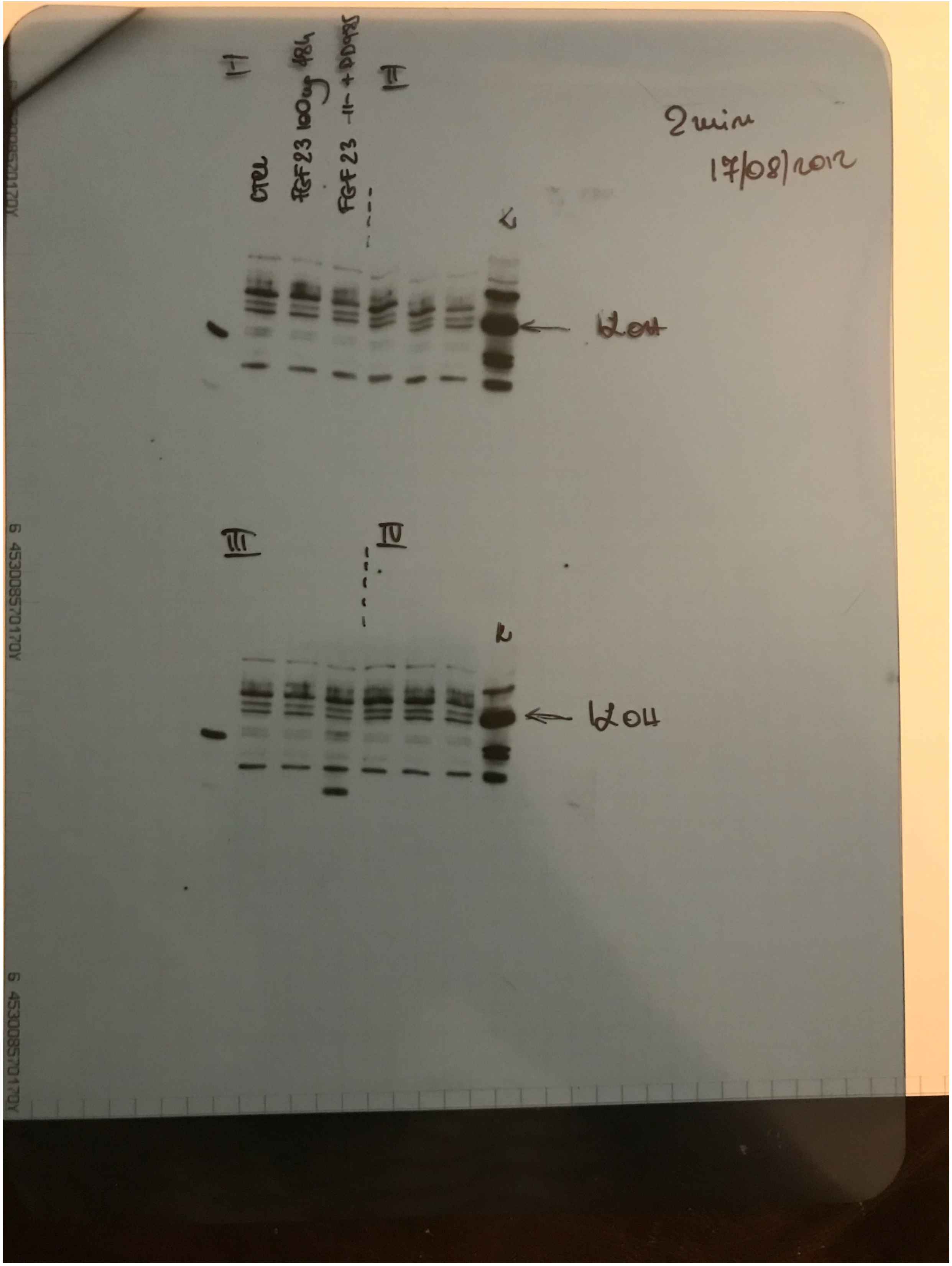

CTRL
FGF23 100ng 48h
FGF23 -II- + PD985
2min
17/08/2012
6 4530085701 70Y

S3 Figure: 24-OHase Western Blot

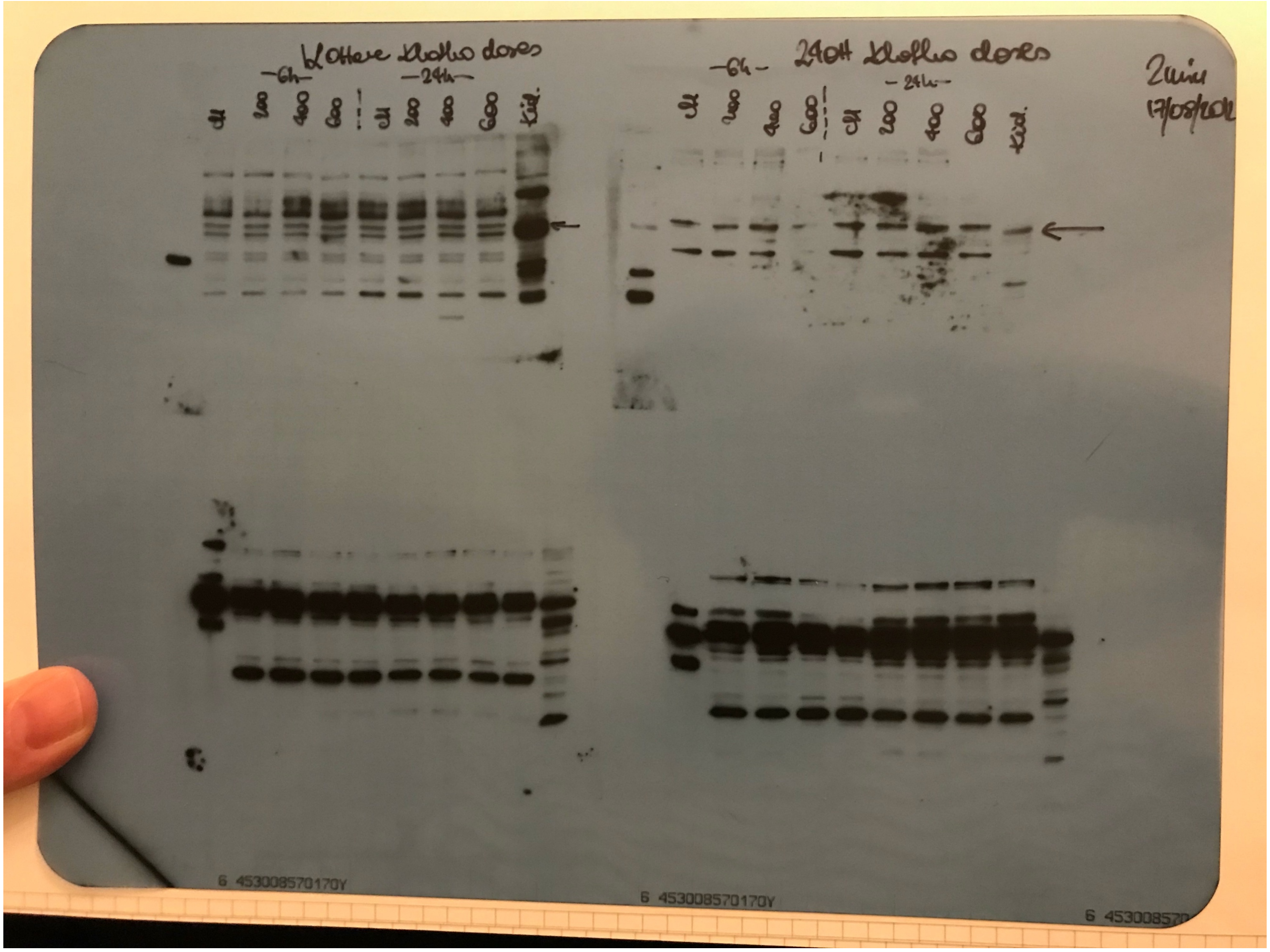

Supplement: S1 Raw images — (PDF) [file pone.0241976.s001.pdf]
